# New criteria for the molecular identification of cereal grains associated with archaeological artefacts

**DOI:** 10.1038/s41598-017-06390-x

**Published:** 2017-07-26

**Authors:** Andre Carlo Colonese, Jessica Hendy, Alexandre Lucquin, Camilla F. Speller, Matthew J. Collins, Francesco Carrer, Regula Gubler, Marlu Kühn, Roman Fischer, Oliver E. Craig

**Affiliations:** 1 BioArCh, Department of Archaeology, University of York, York, YO10 5DD United Kingdom; 2 Department of Archaeology, Max Planck Institute for the Science of Human History, Jena, 07745 Germany; 3 EvoGenomics, Natural History Museum of Denmark, University of Copenhagen, Øster Voldgade 5–7, 1350 Copenhagen, Denmark; 4 McCord Centre for Landscape, School of History, Classics and Archaeology, Newcastle University, Newcastle upon Tyne, NE1 7RU United Kingdom; 5 Archaeological Service of the Canton of Bern, CH-3001 Bern, Switzerland; 6 Integrative Prähistorische und Naturwissenschaftliche Archäologie (IPNA), Spalenring 145, CH-4055 Basel, Switzerland; 7 Target Discovery Institute, University of Oxford, NDMRB, Oxford, OX3 7FZ United Kingdom

## Abstract

The domestication and transmission of cereals is one of the most fundamental components of early farming, but direct evidence of their use in early culinary practices and economies has remained frustratingly elusive. Using analysis of a well-preserved Early Bronze Age wooden container from Switzerland, we propose novel criteria for the identification of cereal residues. Using gas chromatography mass spectrometry (GC-MS), we identified compounds typically associated with plant products, including a series of phenolic lipids (alkylresorcinols) found only at appreciable concentration in wheat and rye bran. The value of these lipids as cereal grain biomarkers were independently corroborated by the presence of macrobotanical remains embedded in the deposit, and wheat and rye endosperm peptides extracted from residue. These findings demonstrate the utility of a lipid-based biomarker for wheat and rye bran and offer a methodological template for future investigations of wider range of archaeological contexts. Alkylresorcinols provide a new tool for residue analysis which can help explore the spread and exploitation of cereal grains, a fundamental component of the advent and spread of farming.

## Introduction

One of the challenges in identifying cereals through organic residue analysis is their relatively low content of chemically stable lipid compounds and high content of carbohydrate (starch) which is rapidly solubilized during cooking and quickly degrades in the burial environment. So whilst edible plants with oil rich seeds and epicuticular leaf waxes have been widely identified in ceramic vessels^1, 2^, reports of cereals are notable by their absence. In some cases, more recalcitrant sterol and terpenoid compounds persist but these rarely offer any taxonomic resolution. One exception is the identification of miliacin in ceramics^3^, a pentacyclic triterpene methyl ether found in broomcorn millet (*Panicum miliaceum*). However, no such biomarkers currently exist for the identification of other cereals, such as maize, foxtail millet, rice and the major Western Eurasia crops of wheat, barley, and rye. Indeed, much of what we know of the history of cereals is derived from macro- and micro-botanical analysis^4–8^, typically charred grains or phytoliths, although this has now been extended by recovery of ancient DNA retrieved from wheat seeds^9, 10^. However, botanical remains in archaeological sites have not always been systematically collected and archived, and their survival may be compromised by cooking practices, food processing and environmental conditions^6, 11, 12^. Furthermore these remains are usually found in contexts associated with plant cultivation, processing and storage rather than consumption. Yet, it is the latter which is particularly relevant to our understanding of the cultural value of these foods, particularly to distinguish their role as dietary staples, in the production of fermented products such as beer and bread, or even as exotic luxuries.

**Figure 1:**
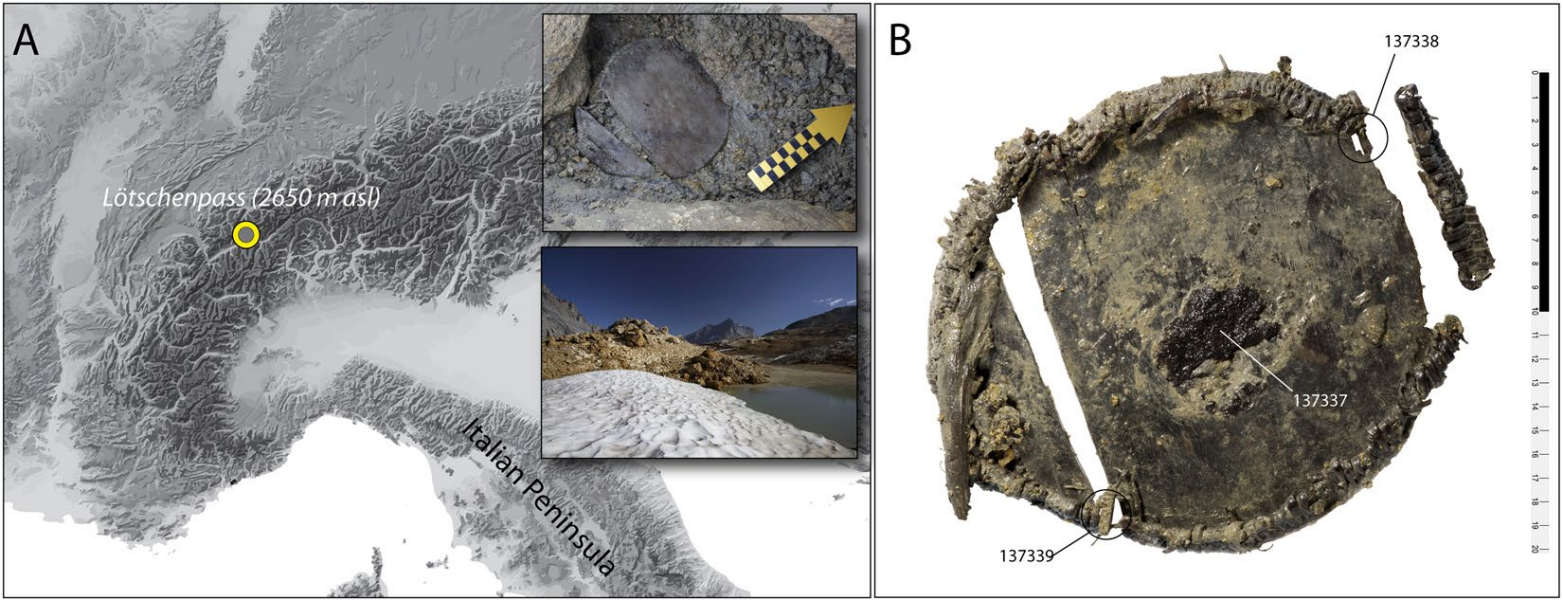
(**A**) geographic and local position of the artefact at the summit of the Lötschenpass (2650 masl); (**B**) the artefact showing the areas sampled for lipid and protein analysis. The map was generated using adobe illustrator CS on an acquired map (Custom map of the Alps from *Map resources* ^®^). The photos were taken by Rolf Wenger, Marcel Cornelissen and Badri Redha (*Archaeological Service of the Canton of Bern*). The copyright holders gave permission to publish the images under Open Access licence (print and digital).

The discovery of an Early Bronze Age wooden container from the summit of the Lötschenpass in Switzerland (Fig. 1A,B) has provided a starting-point for the development of cereal specific biomarkers. The Alpine ice patch in which the artefact was found provides optimum conditions for biomolecular preservation. The wooden vessel contained an amorphous residue on its central surface (#137337; Fig. 1B). A mixture of spelt (*Triticum spelta*), emmer (*Triticum dicoccon*) and barley (*Hordeum vulgare*) could be microscopically observed embedded within the residue, evidenced by numerous fragments of wheat (*Triticum* sp.) testae, wheat pericarp, poorly-preserved spelt and emmer glumes, glume base (*Triticum spelta* and *Triticum dicoccon*), and one segment of barley rachis (*Hordeum vulgare*).

In order to identify lipid components in the preserved residue, we applied gas chromatography mass spectrometry, a technique routinely applied to ceramic artefacts^13, 14^, following sequential extraction with dichloromethane:methanol to obtain a total lipid extract (TLE) and acidified methanol to obtain an acid extract (AE). Lipid analysis was also performed on samples of wood obtained from two extremities of the vessel (#137338, #137339) which served as controls (SI1).

## Results

**Figure 2:**
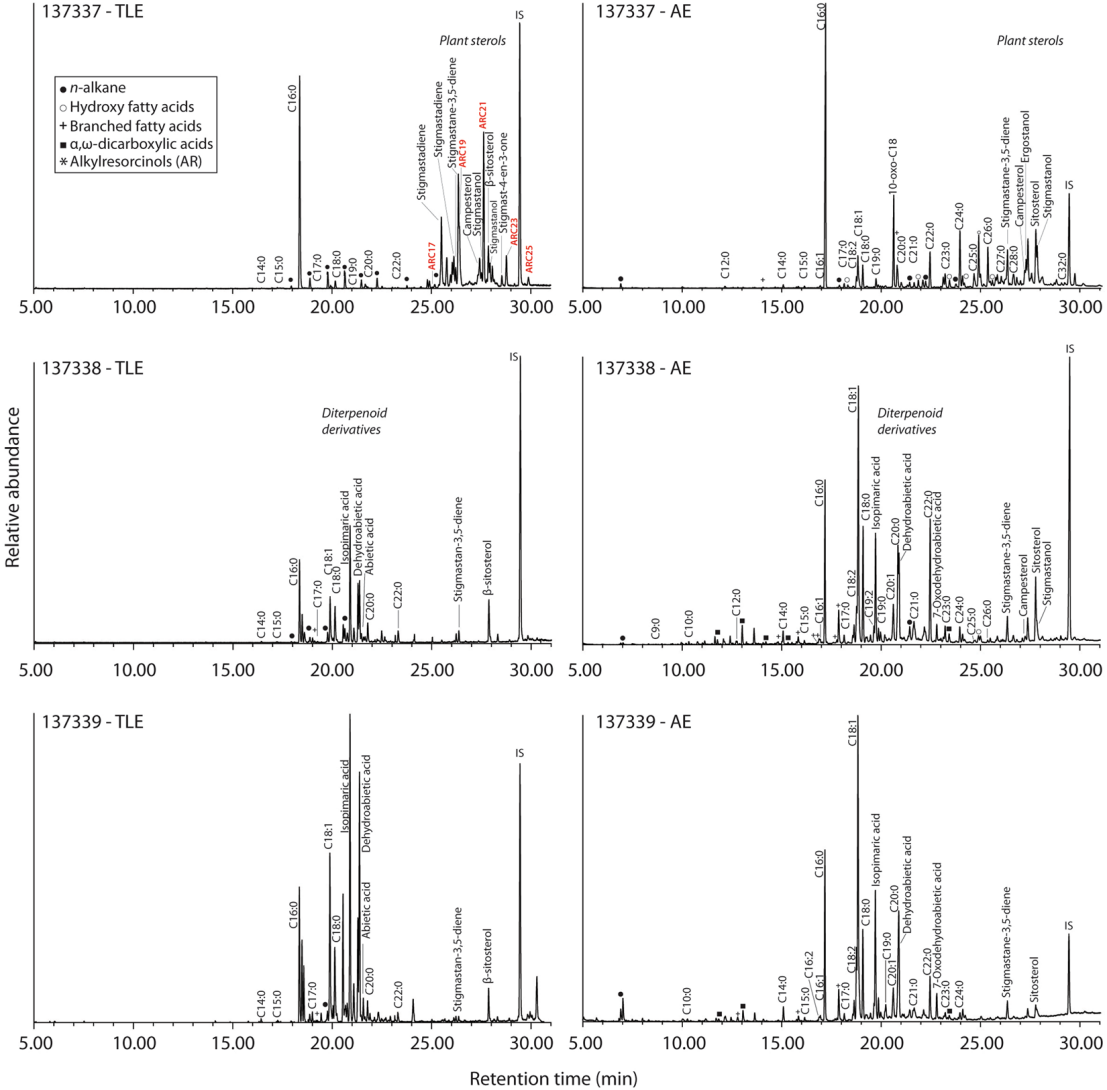
Partial total ion current chromatogram of lipid extracts from the residue (#137337) and the wooden artefact (#137338 and #137339) of total lipid extracts (left) analysed as TMS ethers and acid extracts (right) analysed as methyl esters using a DB-5 ms column. Alkylresorcinols in TLE of the residue are reported in red.

Overall, a greater amount of lipid was recovered from the residue (#137337-AE~18.2 μg mg^−1^, #137337-TLE~12.7 μg mg^−1^) compared to the samples of wood (#137338-AE and #137339-AE~9.4 μg mg^−1^ and 5.5 μg mg^−1^ respectively; #137338-TLE and #137339-TLE~4.0 μg mg^−1^ and ~6.6 μg mg^−1^ respectively). In addition, the distribution of fatty acids differed between the two sample types; the residue contained a range of fatty acids, principally C_16:0_, followed by C_24:0_, C_26:0_ and C_22:0_, while the wood samples had a higher relative concentration of C_18:1_, followed by C_16:0_, C_18:0_ and C_20:0_ (Fig. 2).

Extracts from the residue also contained high concentrations of plant/fungal sterols, particularly in the acid extracts (AE), including campesterol, ergostanol, β-sitosterol, stigmastanol, and degradation products of phytosterols (stigmastadiene, stigmastane-3,5-diene^15^). Cholesterol and cholesterol derivatives were absent and consistent with a predominantly plant origin of the residue. The presence of 10-oxo-octadecanoic acid (10-oxo-C_18:0_; Fig. 2A) as detected as its methyl ester in the AE (m/z **312**, 156, 281, 214, 182) is most likely explained by microbial oxidation of C_18:1_^16^. In contrast, the wooden container contained coniferous resin, including predominantly isopimaric acid and abietic acid, along with degradation products due to aromatisation or oxidation, such as 7-oxodehydroabietic acid and dehydroabietic acid^17^. These diterpenoid derivatives were not detected in the residue and therefore are most likely to be endogenous to the coniferous wood used to manufacture the container.

**Figure 3:**
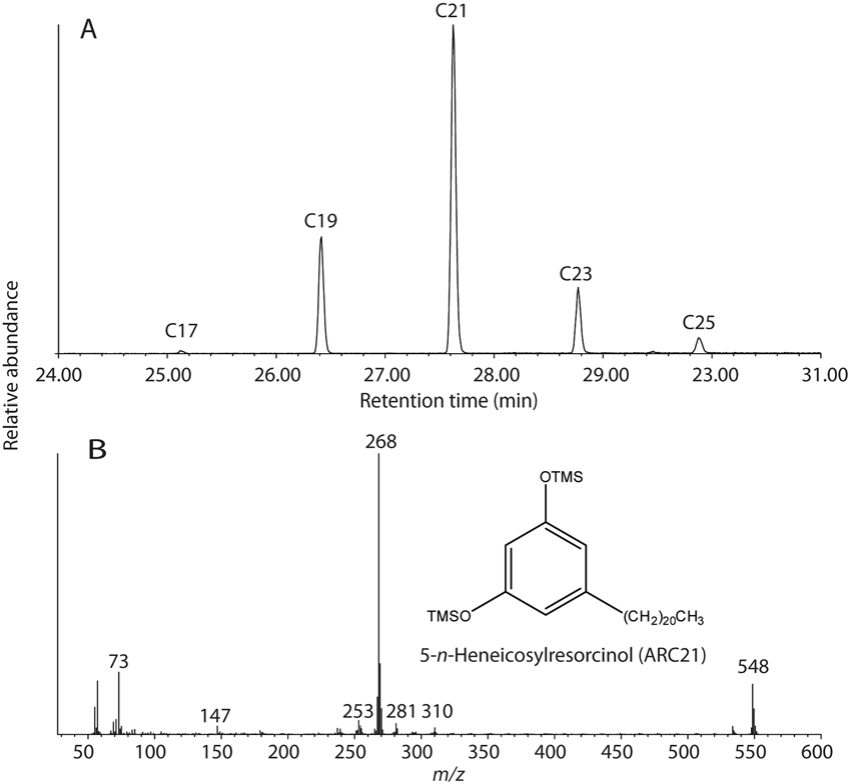
(**A**) Partial extracted ion chromatogram (m/z 268) showing homologues of alkylresorcinols (1,3-dihydroxy-5-alkylbenzenes) with alkyl chains of 17 to 25 carbon atoms in an extract of sample #137337-TLE; (**B**) selected mass spectra and structural information for 5-n-Heneicosylresorcinol TMS ester containing an alkyl chain of 21 carbon atoms.

Of particular relevance is the identification of a class of phenolic lipids, 1,3-dihydroxy-5-alkylbenzenes or alkylresorcinols (ARs) observed in the trimethylsilylated solvent extract (TLE) of the residue (Figs 2B and 3A,B). Alkylresorcinols consist of an odd number of carbon atoms, typically C_17_ to C_25_, in their alkyl chain, and are at high concentrations in the bran fraction of wheat and rye, although they are also found in lesser quantities in barley, millet, and maize bran^18–21^. Whilst some bacteria also produce ARs^22–24^, a bacterial origin is much less likely given the absence of other bacteria derived lipid in the residue and the absence of ARs in the available control samples. Instead, the high relative concentrations of ARs in the residue (~1.4 μg mg^−1^, 1.5% of the TLE) and their association with other plant derived lipids strongly suggests that these compounds are derived from cereal grain.

Previous studies have shown that the ratio of the AR homologues C_17:0_ and C_21:0_ (C_17:0_/C_21:0_) differ markedly between wheat and rye^18, 19^, with much higher relative amounts of C_17:0_ in rye and that these ratios are not altered by food processing^18^. ARs in the surface deposit were present with alkyl chains from C_17:0_ to C_25:0_, but with a higher concentration of C_19:0_ and C_21:0_ homologues and a C_17:0_/C_21:0_ of 0.02, closer to the value for wheat (C_17:0_/C_21:0_~0.1), compared to rye (C_17:0_/C_21:0_~1.0). This seems to indicate that wheat grain was the main source of these lipids, but we cannot exclude diagenetic alteration to the ratio of C_17:0_/C_21:0_, which should be assessed in the future through simulated degradation experiments. Interestingly, we found no evidence of compounds formed from the heating of fatty acids at higher temperatures, such as long-chain ketones^25^ or ω-(o-alkylphenyl) alkanoic acids (APAAs)^26^. Therefore the grains were probably not subject to protracted heating, although it is thought that the isomerisation reaction needed to form the APAAs requires the presence clay minerals within ceramic^26^. We are therefore confident in assigning the ARs attribution to wheat, which is broadly supported by the protein analysis. Finally, we note that ARs were not detected in the AE of the residue when derivatised to produce any trimethylsilyl ethers indicating that this commonly used screening approach is not suitable for detecting these compounds.

To supplement this analysis and provide greater taxonomic resolution, we explored a shotgun proteomics approach to assess the survival of proteins. Although the application of proteomics to pottery is still in its infancy, both animal^27^, and plant proteins^28^ have been recovered, even though some of the reported sequences are rather surprising e.g. ref. 29. Frustratingly, many biochemical products used in the laboratory are derived from ‘foods’ (e.g. milk, blood) and may appear as common laboratory contaminants, and the assignment to a parent protein needs to be carefully considered. The combination of lipid and proteomic approaches to the same sample, provides a potential means of independently verifying protein survival. Protein extraction and analysis of the amorphous residue yielded a total of 47 confidently identified proteins, with 27 of plant origin, 6 common laboratory contaminants or experimentally derived proteins (keratins and trypsin, respectively) and 14 proteins of microbial or highly conserved origin. No milk or meat derived proteins were detected, consistent with the results from extracted fatty acids.

Of the 27 confidently identified plant proteins, all but one were taxonomically assigned to grasses (SI2). More specifically, we were able to identify proteins belonging to the Triticeae tribe, and *Triticum* genus. In addition, protein basic endochitinase A matches taxonomically to the subtribe Hordeinae, a subtribe of Triticeae which consists of barley and rye (*Hordeum* sp. and *Secale* sp. respectively). The presence of these taxonomically distinctive peptides may indicate the presence of at least two grain types; wheat (*Triticum﻿﻿* sp.) and a member of the Hordeinae subtribe (i.e. Barley/Rye). Moreover, many of the identified proteins were tissue specific, with their expression being localised to the endosperm (grain) of the plant (SI2). Comparing our results to the analysis of preserved foodstuffs by Shevchenko *et al*.^28^, some similar proteins, although different species, were detected, including serpins, xylanase and chymotrypsin inhibitors and oxalate oxidase. Looking at the functions of these identified proteins, the majority are related to plant defence, such as endochitinase, serpins, and amylase inhibitors. Quantitative whole proteome studies of developing wheat grains suggest that proteins related to defence are highly expressed at the mature stage of grain development^30, 31^. While the analysis in this study is not quantitative, the dominant presence of defence proteins could indicate that this plant-based residue is derived from cereals which were collected as mature, ripe grains. It should be noted that common domesticated species are well represented in reference sequence databases, and therefore other potential, unrepresented Hordeinae taxa would not be detectable by this approach. However, the protein results from this study are supported by the lipid and archaeobotanical analyses, providing compelling evidence for the presence of grain cereals in the vessel.

## Discussion

Although rarely preserved, the use of perishable containers, such as baskets or barrels, for the storage of cereals has been previously suggested in relation to the large accumulation of cereals grains frequently found in Bronze Age cave sites in the European Alps and surrounding areas^5^. These are thought to have been short-term repositories for storing grains, including millets, barley and wheats. Splint boxes are very rare and the only comparable artefact was found on another pass, the Schnidejoch, 25 km to the West^32^. Neither of the artefact assemblages found on these two mountain passes includes pottery vessels, suggesting splint boxes and containers made of leather were the preferred method to carry provisions and equipment. This is also supported by the two bark containers associated with the Iceman found in the Tissenjoch (South Tyrol, Italy)^33^. In crossing high Alpine passes, the weight of the equipment was – and still is – of considerable importance.

The Lötschenpass, along with a number of other passes in the Bernese Alps, connects the Western Swiss Plateau to the Valais valley, which in turn connects with the Italian Peninsula. A number of lake shore pile dwellings existed on the Swiss Plateau during the Early Bronze Age, while in the Valais the settlement record is thinner but the large number of Early Bronze graves show that the valley was not only settled but people imported goods from north and south of the Alps^34^. In this context, the wooden container found on the Lötschenpass can be linked with either trading connections or seasonal movements from lowland areas to upland pastures as part of the pastoral economy, although hunting could also explain the requirement to access such rocky and glaciated areas of the high Alps^32^. We note that a biomarker for broomcorn millet (miliacin^3^) was not identified in the residue. Further work utilizing different cereal biomarkers together with any archaeobotanical evidence, could be applied to understand the seasonal use of these passes, as millets appear in botanical assemblages in Europe from the 3rd Millennium BC^35^ but are likely to be harvested at a different times of the year compared to wheat and barley.

Our findings also raise important issues regarding the absence of evidence for cereals in pottery associated with ancient agricultural economies. Despite the thousands of ceramic artefacts submitted for lipid residue analysis, the evidence for cereals is extremely limited^3^. This fact is particularly intriguing as we know that cereals were of major economic importance since the emergence of farming and that they generally require extensive processing prior to consumption, which must have involved various types of artefacts, including pottery. However, the organic residue analysis so far undertaken suggests Bronze Age pots are largely associated with the processing of animal products^36^, similar to findings from the extensive analysis of Neolithic ceramic vessels^37^. Whether this difference is attributable to generally poor preservation of molecular markers for cereal grains, such as the alkylresorcinols presented here, or due to real differences in the types of vessels used to store and process animal and plant foods, requires further analysis and experiments aimed at simulating use and degradation.

## Methods

### Geographic and cultural setting

The wooden vessel was discovered in 2012 near the summit of the Lötschenpass (2650 m asl), in the western Bernese Alps (Switzerland) (Fig. 1A,B). Lötschenpass, together with the Schnidejoch pass, is one of the few sites in the Alps that have produced archaeological finds from melting ice^32^. These passes connect the Swiss Plateau with the Valais valley and ultimately Italy. In the northern valleys leading up to the passes and in the Valais, numerous graves and a few settlement sites dated to the Late Neolithic and Early Bronze Age are known^32, 34, 38, 39^. The wooden vessel has a circular shape, with its round base made of Swiss pine (*Pinus cembra*), and the bent rim made of willow (*Salix* sp.), sewn together with splint twigs of European larch (*Larix decidua*). A direct ^14^C age of 1940–1755 cal BC (ETH-58204, 3527 ± 32 years BP) was obtained on a piece of the binding, confirming its Early Bronze Age attribution. An almost identical Early Bronze Age object was found in the Schnidejoch pass (2756 m asl), located east of the Lötschenpass^32^.

The wooden vessel preserved an amorphous residue on its central surface (#137337; Fig. 1B), that was sampled for microscopic, lipid and protein analyses. Lipid analysis was also performed on samples of wood obtained from two extremities of the vessel (#137338, #137339).

### Archaeobotanical analysis

Microscopic analysis were performed using magnifications from 6.3 to 400x under binocular microscope following criteria described in refs 40, 41.

### Lipid extraction and derivatization

Samples for lipid analysis were removed from three areas of the artefact (Fig. 1B), two directly from the wooden surface at the extremities (#137338, #137339) and one from the residue preserved at the centre of the container (#137337), and freeze-dried. Subsamples of each - 137337 (~14.2 mg), 137338 (~19.05 mg), 137339 (~30.5 mg) - were extracted using 2:1 DCM:MeOH (3 × 2 mL) to produce a total lipid extract (TLE). An aliquot of solvent (50% vol/vol) was removed to a clean vial, dried under N_2_ and derivatized with N,O-bis(trimethylsilyl)trifluoroacetamide (BSTFA) +1% TMC (70 °C for 1 h) to produce trimethylsilyl (TMS) ethers. To maximise recovery and release any bound lipid, the remaining contents (sample and solvent) was sequentially treated with acidified methanol to produce an acid extract (AE)^14, 42^. Briefly the sample was dried under a gentle stream of N_2_, then 2 mL of methanol were added. The suspended fraction ultrasonicated for 15 min and 400 μL of H_2_SO_4_ was added and tubes were heated at 70 °C for 2 h to produce methyl esters. The supernatant was extracted with hexane (3 × 2 mL) and neutralised with K_2_CO_3_. A separate aliquot of the AE of the residue (#137337) was treated with BSTFA as above and analysed separately.

Total lipid and acid extracts were dried under a gentle stream of N_2_ and an internal standard (10 μg hexatriacontane) added to each sample before analysis by GC using an Agilent 7890A gas chromatograph (Agilent Technologies, Cheadle, Cheshire, UK). The injector was splitless and maintained at 300 °C and injected 1 µL of sample into the GC. The column used was a 100% Dimethylpolysiloxane DB-1 (15 m × 320 µm × 0.1 µm; J&W Scientific, Folsom, CA, USA). The carrier gas was hydrogen with a constant flow rate of 2 ml/min. The temperature program was set at 100 °C for 2 min, rising by 20 °C/min until 325 °C. This temperature was maintained for 3 min. The total run time was 16.25 min. The lipids from both extracts were quantified according to the internal standard and diluted appropriately prior to GC-MS.

### Gas chromatography-mass spectrometry (GC-MS)

GC-MS was performed on both AE and TLE using a 7890A Series chromatograph attached to a 5975C Inert XL mass-selective detector with a quadrupole mass analyser (Agilent Technologies, Cheadle, UK). The carrier gas used was helium, and the inlet/column head-pressure was constant. A splitless injector was used and maintained at 300 °C. The GC column was inserted directly into the ion source of the mass spectrometer. The ionisation energy of the mass spectrometer was 70 eV and spectra were obtained by scanning between m/z 50 and 800. Two different column phases were used. General screening of both AE and TLE was performed using a DB-5 ms (5%-phenyl)-methylpolysiloxane column (30 m × 0.250 mm × 0.25 μm; J&W Scientific, Folsom, CA, USA). The temperature for this column was set at 50 °C for 2 min, then raised by 10 °C/min to 325 °C, where it was held for 15 min. The TLE was also analyzed with a HT-DB1, 100% Dimethylpolysiloxane (15 m × 0.320 mm × 0.1 µm) (J&W Scientific, Folsom, CA, USA). The injector was maintained at 350 °C. The temperature of the oven was set at 50 °C for 2 min, and then raised by 10 °C min^−1^ to 350 °C, where it was held for 15 min.

### Protein extraction and nLC-MS/MS

To further elucidate the composition of the residue (#137337) a ‘shotgun’ proteomic analysis was performed. Tryptic peptides were extracted using a protocol based on GASP^43^, modified for ancient samples. 4.0 mg of sample #137337 was ground using a sterile micropestle and 5 μL of SDS (20%) added with 45 μL of M-PER (Mammalian Protein Extraction Reagent, Thermo Fisher), then shaken for 15 min at room temperature. 50 μL of DTT (1 M) was added and the sample shaken for a further 30 min at room temperature. 100 μL of Proto-Gel (37.5:1 Acrylamide to Bisacrylamide, National Diagnostics) was added and gently resuspended to mix, then left for 20 min. To polymerize the gel, 8 μL of tetramethylethylenediamine (TEMED), followed by 8 μL of ammonium persulfate (APS) was added and gently mixed. The polymerized gel was then shredded to increase the surface area by passing the gel through a plastic grid insert by pulse centrifugation. The gel pieces were fixed through the addition of methanol/water/acetic acid solution (50/40/10). The solution was centrifuged, and then the supernatant discard. 1 mL of acetonitrile was added to dehydrate the gel pieces. 1 mL of urea (6 M) was added to the dehydrated gel pieces and rotated for 3 min. A series of washing and drying steps to exchange buffers and remove salts was then performed. For protein digestion, samples were incubated overnight at 37 °C in 200 μL of ammonium bicarbonate (0.05 M) and trypsin (5 μL of 0.5 μg/μL).

Following trypsin digestion, the gel pieces were dried with acetonitrile to extract peptides and the supernatant transferred into a fresh Eppendorf tube. 200 μL of 5% formic acid solution, followed by another drying step with acetonitrile, was added to the gel pieces to extract acidic peptides, and the supernatant transferred. To fully dehydrate the gel and remove remaining peptides, a further 200 μL of acetonitrile was added, rotated for 5 min, pulse centrifuged, and the supernatant transferred. The extracted peptides were then dried in a centrifugal evaporator, and desalted using C18 resin ZipTips (EMD Millipore) prior to MS/MS analysis.

Tandem Mass Spectrometry was performed on an Orbitrap Fusion Lumos (Thermo Fisher) at the Mass Spectrometry Laboratories of the Target Discovery Institute at the University of Oxford. Preceding injection into the nLC-MS/MS, dried peptides were resuspended in 20 μL of 0.1%Trifluoroacetic acid and 2% acetonitrile, and 6 μL injected. Chromatographic peptide separation was achieved using a 50 cm easy spray column (Thermo Scientific) and a linear Acetonitrile gradient from 2–35% in 5% DMSO and 0.1% formic acid. Precursor peptides were detected with up to 50 ms accumulation time for an ion target of 4E5, followed MS/MS data acquisition for up to 3 seconds and a maximum parallel injection time of 250 ms per precursor mass. Precursors were isolated in the quadrupole with 1.2 Th and selected at an intensity of 5000 or higher following automatic exclusion for 60 seconds. MS1 spectra were acquired with a resolution of 120.000, while MS/MS spectra were acquired after CID fragmentation (35% collision energy) in the linear ion trap in rapid scan mode.

### MS/MS data analysis

Raw spectral data were converted to Mascot generic format (mgf) using Proteowizard MSConvert (version 3.0.4743) using the 100 most intense peaks in each MS/MS spectrum. MS/MS ion database searching was performed on Mascot (Matrix Science^TM^, version 2.4.01), against the UniProt database. Searches were performed against a decoy database to estimate a false discovery rate (FDR), which was subsequently adjusted to 1%. Propionamide (C) was set as a fixed modification and deamidation (NQ), methionine oxidation, propionamide (K) and propionamide (N-terminus) were set as variable modifications. Peptide tolerance was 10 ppm, and MS/MS ion tolerance was 0.5 Da. Semi-tryptic peptides were searched with up to 2 missed cleavages. Results were adjusted to a 1% FDR and filtered to an ion score of >35, with protein identifications requiring a minimum of two peptides. Peptides identified by Mascot as being in the kingdom Plantae were aligned using BLASTp against all non-redundant nucleotide sequences and the taxonomy assessed using the BLAST Lineage Report. Protein expression data was retrieved, where available, from UniProtKB.

### Data Availability

Mass spectrometry data has been deposited to the ProteomeXchange Consortium^44^ via the PRIDE partner repository under accession code PXD005908.

## Electronic supplementary material

Supplementary Information
