# Supplementary material for: New criteria for the molecular identification of cereal grains associated with archaeological artefacts
Source: Sci Rep. 2017 Jul 26;7:6633. doi: 10.1038/s41598-017-06390-x (PMC5529501; doi:10.1038/s41598-017-06390-x)
Supplement: Supplementary file 1 — Supplementary Information [file 41598_2017_6390_MOESM1_ESM.pdf]

New criteria for the molecular identification of cereal grains associated with archaeological artefacts

Andre Carlo Colonese[1], Jessica Hendy[1,2], Alexandre Lucquin[1], Camilla Speller[1], Matthew Collins[1,3], Francesco Carrer[4], Regula Gubler[5], Marlu Kühn[6], Roman Fischer[7],Oliver E. Craig[1]

1. BioArCh, Department of Archaeology, University of York, York, YO10 5DD, United Kingdom.
2. Department of Archaeology, Max Planck Institute for the Science of Human History, Jena, 07745, Germany
3. EvoGenomics, Natural History Museum of Denmark, University of Copenhagen, Øster Voldgade 5-7, 1350 Copenhagen, Denmark
4. McCord Centre for Landscape, School of History, Classics and Archaeology, Newcastle University, Newcastle upon Tyne, NE1 7RU, United Kingdom
5. Archaeological Service of the Canton of Bern, CH-3001, Bern, Switzerland
6. Integrative Prähistorische und Naturwissenschaftliche Archäologie (IPNA), Spalenring 145, CH-4055, Basel, Switzerland
7. Target Discovery Institute, University of Oxford, NDMRB, Oxford, OX3 7FZ

Supplementary information

Sample	Extraction	Lipid detected	Attribution
**137337**	AE	FA (C_{12}-C_{32}, $C_{16:1}$-$C_{18:1}$, C_{17br}-C_{20br}), ALK (C_{11}, C_{20}-C_{29}), HFA (C_{16}, $C_{20, 22-25}$), Stigmastane-3,5-diene, campesterol, ergostanol, β-sitosterol, stigmastanol, 10-oxo-$C_{18:0}$	Plant
**137338**	AE	FA (C_9-C_{26}, $C_{16:1}$-$C_{20:1}$, C_{14br}-C_{17br}), ALK (C_{11}, C_{23}), HFA (C_{24}), Stigmastane-3,5-diene, campesterol, β-sitosterol, stigmastanol, DT, DC (C_{8-11}, C_{20})	Plant (conifer)
**137339**	AE	FA (C_{10}-C_{24}, $C_{16:1,2}$-$C_{20:1}$, C_{15br}-C_{17br}), ALK (C_{11}), Stigmastane-3,5-diene, β-sitosterol, DT, DC (C_{8-9}, C_{20})	Plant (conifer)
**137337**	TLE	FA (C_{14}-C_{22}), ALK (C_{20}-C_{29}), Stigmastadiene, Stigmastane-3,5-diene, campesterol, stigmastanol, β-sitosterol, stigmast-4-en-3-one, AR (C_{17}-C_{25})	Cereal
**137338**	TLE	FA (C_{14}-C_{22}, C18:1, C17br), ALK (C_{20}-C_{24}), Stigmastane-3,5-diene, β-sitosterol, DT	Plant (conifer)
**137339**	TLE	FA (C14-C22, C18:1, C17br), ALK (C_{23}), Stigmastane-3,5-diene, β-sitosterol, DT	Plant (conifer)

SI1. Samples selected for GCMS and details of the absorbed lipids detected. FA (Cx:y) - fatty acids with carbon length x and number of unsaturations y, br -branched chain acids; DC - α,ω-dicarboxylic acids; ALK (Cx) - n-alkanes with carbon length x; HFA – Hydroxy fatty acids; 10-oxo-$C_{18:0}$ - 10-oxo-octadecanoic acid; DT, Diterpenoid derivatives - isopimaric acid, abietic acid, 7-oxodehydroabietic acid and dehydroabietic acid. AR(Cx) - alkylresorcinols with carbon length x.

Protein	Species	Common name	Function	Mascot Score	Peptide	BLAST Results (Lowest Common Ancestor)
Basic endochitinase C and Basic endochitinase A*	*Hordeinae*	Barley/Rye	Defence	283	R.YCDILR.V	Not unique to plants
					R.AIGVDLLR.N	Not unique to plants
					Y.CFKQER.G + Deamidated (NQ)	Not unique to plants
					R.AIGVDLLRN.P	Mesangiospermae
					R.AIGVDLLRN.P + Deamidated (NQ)	Mesangiospermae
					P.GFGTTGSTDTR.K	Matches to *S. cereale*, *H. bulbosum* & *T. aesitvum*
					R.AAGRVPGFGVITN.I	Pentapetalae
					N.GGIECGHGQDSR.V	Pooideae
					P.DLVATDPTVSFK.T	Mesangiospermae
					A.GAFPGFGTTGSTDTR.K	Matches *S. cereale* & *T. aestivum*
					H.AVITGQWSPSGTDR.A + Deamidated (NQ)	Triticeae
					R.NPDLVATDPTVSFK.T	Mesangiospermae
					N.IVNGGIECGHGQDSR.V	Pooideae
					N.IVNGGIECGHGQDSR.V	Pooideae
					N.IVNGGIECGHGQDSR.V + Deamidated (NQ)	Pooideae
					N.IINGGLECGHGQDSR.V	Pooideae
					N.IINGGLECGHGQDSR.V	Pooideae
					N.IINGGLECGHGQDSR.V + Deamidated (NQ)	Pooideae
					T.NIVNGGIECGHGQDSR.V	Pooideae
					F.VAAAGAFPGFGTTGSTDTR.K	Matches *S. cereale* & *T. aestivum*
					D.LLRNPDLVATDPTVSFK.T	Hordeinae
					G.VGYGDNLDCYNQRPFA.-	Mesangiospermae
					G.VGYGDNLDCYNQRPFA.- + Deamidated (NQ)	Mesangiospermae
					R.VGYGNNLDCYNQRPFA.- + 2 Deamidated (NQ)	Mesangiospermae

					L.GVGYGDNLDCYNQRPFA.-	Mesangiospermae
					R.GATSNYCTPSAQWPCAPGK.S + Deamidated (NQ)	Hordeinae
					R.GATSNYCTPSAQWPCAPGK.S + 2 Deamidated (NQ)	Hordeinae
					R.AIGVDLLRNPDLVATDPTVSFK.T	Hordeinae
					R.AIGVDLLRNPDLVATDPTVSFK.T	Hordeinae
					R.AIGVDLLRNPDLVATDPTVSFK.T + Deamidated (NQ)	Hordeinae
					R.AIGVDLLRNPDLVATDPTVSFKTA + Deamidated (NQ)	Hordeinae
					R.YCDILGVGYGDNLDCYNQRPFA.-	Triticeae
					R.YCDILGVGYGDNLDCYNQRPFA.- + Deamidated (NQ)	Triticeae
					R.YCDILGVGYGDNLDCYNQRPFA.- + 2 Deamidated (NQ)	Triticeae
					R.YCDILGVGYGDNLDCYNQRPFA.- + Deamidated (NQ)	Triticeae
					A.AGRVPGFGVITNIVNGGIECGHGQDSR.V + 2 Deamidated (NQ); Propionamide (N-term)	Triticeae
Probable aquaporin TIP3-2	BOP clade	Grasses	Facilitates water transport	169	N.ILAGGPFDGAAMNPAR.A + Oxidation (M)	BOP clade
					A.NILAGGPFDGAAMNPAR.A	Poaceae
					A.NILAGGPFDGAAMNPAR.A + Oxidation (M)	Poaceae
					A.GANILAGGPFDGAAMNPAR.A + Oxidation (M)	Poaceae
Serpin-Z1 A/B/C*	*Triticum aestivum*	Wheat	Defence	154	R.LSIAHQTR.F	Not unique to plants
					R.LASTISSNPK.S	*Triticum/Aegilops*
					Q.GTEAAASTAIK.M	*Triticum/Aegilops*
					K.LSAEPDFLER.H	*Triticum/Aegilops*
					R.EDISGVVLFMGH.V	*Triticum/Aegilops*
					R.EDISGVVLFMGH.V + Oxidation (M)	*Triticum/Aegilops*
					K.YKADTQSVDFQTK.A + Deamidated (NQ)	*Triticum aestivum*
					F.VEVNEQGTEAAASTAIK.M	*Triticum/Aegilops*
					F.MSSMDDQYLSSSDGLK.V + Oxidation (M)	Matches *T. urartu* & *T.*

						aestivum
					A.FVEVNEQGTEAAASTAIK.M + Propionamide (N-term)	Matches *T. urartu* & *T. aestivum*
					Q.AFVEVNEQGTEAAASTAIK.M + Deamidated (NQ)	*Triticum/Aegilops*
					Q.AFVEVNEQGTEAAASTAIK.M	*Triticum/Aegilops*
					R.VSSVFHQAFVEVNEQGTEAAASTAIK.M	*Triticum/Aegilops*
Serpin Z2*	*Triticum*	Wheat	Defence	87	R.LSIAHQTR.F	Not unique to plants
					R.LVLGNALYFK.G	Poaceae
					R.LVLGNALYFK.G + Deamidated (NQ)	Poaceae
					R.EDTSGVVLFIGH.V	*T. aestivum, urartu & Aegilops*
					A.FVEVNETGTEAAATTIAK.V + Propionamide (N-term)	*T. aestivum, urartu & Aegilops*
					K.AFVEVNETGTEAAATTIAK.V + Deamidated (NQ)	*T. aestivum, urartu & Aegilops*
Alpha-amylase/trypsin inhibitor CM3*	*Triticum*	Wheat	Defence	134	R.EMQWDFVR.L	*T. aestivum & durum*
					R.YFIALPVPSQPVD.P	*T. aestivum/Aegilops & durum*
					N.VGESGLIDLPGCPR.E	*T. aestivum/Aegilops & durum*
					R.LLVAPGQCNLATIH.N	*T. aestivum/Aegilops & durum*
					R.YFIALPVPSQPVDPR.S	*T. aestivum/Aegilops & durum*
					R.YFIALPVPSQPVDPR.S + Deamidated (NQ)	*T. aestivum/Aegilops & durum*
					R.SGNVGESGLIDLPGCPR.E	*T. aestivum/Aegilops & durum*
					R.SGNVGESGLIDLPGCPR.E + Deamidated (NQ)	*T. aestivum/Aegilops & durum*
					K.LYCCQELAEISQQCR.C	*T. aestivum & durum*
Alpha-amylase inhibitor 0.53*	*Triticeae*	Wheat	Defence	119	K.LQCNGSQVPEAVLR.D	Triticeae
					K.LQCNGSQVPEAVLR.D + Deamidated (NQ)	Triticeae
					K.LQCNGSQVPEAVLR.D + 2 Deamidated (NQ)	Triticeae
					R.LPIVVDASGDGAYVCK.D	Triticeae
Probable aquaporin PIP2-1	BOP clade	Grasses	Facilitate	114	Y.TVFSATDPKR.N	Embryophyta

			water transport		A.TIPITGTGINPAR.S	Embryophyta
					R.YGGGANTLAAGYSK.G	BOP clade
					R.YGGGANTLAAGYSK.G + Deamidated (NQ)	BOP clade
					H.LATIPITGTGINPAR.S	Embryophyta
Xylanase inhibitor protein 1	*Triticum/Aegilops*	Wheat	Defence	94	R.SALDLFDHL.W	Triticeae
					K.TGQVTVFWGR.N	Poaceae
					R.CGYPPAAHVGR.A	Triticeae
					R.CGYPPAAHVGR.A	Triticeae
					K.NVYYGVAPVAQK.K	*Triticum/Aegilops*
					K.DNYGGIMLWDR.Y	Triticeae
					K.NVYYGVAPVAQKK.D	*Triticum/Aegilops*
					L.SIGGYGTGYSLPSNR.S	Triticeae
Wheatwin 1/2*	*T. aestivum, urartu & Aegilops*	Wheat	Defence	93	N.PATGAQITAR.I	Triticeae
					N.PATGAQITAR.I + Propionamide (N-term)	Triticeae
					R.VTNPATGAQITAR.I	Triticeae
					R.LSIAHQTR.F	Not unique to plants
					R.LVLGNALYFK.G	Poaceae
					R.LVLGNALYFK.G + Deamidated (NQ)	Poaceae
					R.EDTSGVVLFIGH.V	*T. aestivum, urartu & Aegilops*
					A.FVEVNETGTEAAATTIAK.V + Propionamide (N-term)	*T. aestivum, urartu & Aegilops*
					K.AFVEVNETGTEAAATTIAK.V + Deamidated (NQ)	*T. aestivum, urartu & Aegilops*
ADP, ATP carrier protein, mitochondrial	Poaceae	Grasses	ADP/ATP carrier	78	R.GNTANVIR.Y	Not unique to plants
					R.GNTANVIR.Y + Deamidated (NQ)	Not unique to plants
					R.AIAGAGVLSGYDQLQIL.F	Poaceae
					A.LGWLITNGAGLASYPIDTVR.R + Deamidated (NQ); Propionamide (N-term)	Mesangiospermae
Agglutinin isolectin 1 / Agglutinin isolectin 3*	*Triticum*	Wheat	Carbohydrate binding	75	R.VCTNNYCCSK.W	Matches *T. urartu, durum & aestivum*
					K.WGSCGIGPGYCGAGCQSGGCD.G	Triticeae
					K.WGSCGIGPGYCGAGCQSGGCDG.V	Triticeae
					K.WGSCGIGPGYCGAGCQSGGCDG.V + Deamidated (NQ)	Triticeae

Glucan endo-1,3-beta-glucosidase GI*	*Triticeae*	Wheat	Defence	75	R.FDAVTNT.F + Deamidated (NQ)	Not unique to plants
					R.VVVSESGWPSASGFAATADNAR.A	Triticeae
Gamma-gliadin B*	*Triticeae*	Wheat	Major seed storage protein	75	G.IIQPQQPAQLEVIR.S	Triticeae
					Q.GIIQPQQPAQLEVIR.S	Triticeae
					Q.GIIQPQQPAQLEVIR.S + Deamidated (NQ)	Triticeae
Chymotrypsin inhibitor WCI*	*Triticum/Aegilops*	Wheat	Defence	70	R.ELAAISSNCR.C + Deamidated (NQ)	*Triticum/Aegilops*
					R.TLALPGQCNLPTIHGGPY.C	*Triticum/Aegilops*
					A.FPPSQSQGGGPPQPPLAPR.C + Deamidated (NQ); Propionamide (N-term)	*Triticum/Aegilops*
Trypsin/alpha-amylase inhibitor CMX1/ CMX2/CMX3*	*T. aestivum, urartu & Aegilops*	Wheat	Defence	64	R.EFIAGIVGR.E	Not unique to plants
					R.QTCGYYLSAER.Q	*T. aestivum, urartu & Aegilops*
					R.QTCGYYLSAER.Q + Deamidated (NQ)	*T. aestivum, urartu & Aegilops*
Alpha-amylase/trypsin inhibitor CM16*	*Triticeae*	Wheat	Defence	58	R.EVQMDFVR.I	Triticeae
					R.DYVEQQACR.I + Deamidated (NQ)	Triticeae
					K.SRPDQSGLMELPGCPR.E	Triticeae
					K.QQCCGELANIPQQCR.C	Triticeae
					K.QQCCGELANIPQQCR.C + Deamidated (NQ); Propionamide (N-term)	Triticeae
					R.ILVTPGYCNLTTVHNTPYCLAMEE.S + Oxidation (M)	*T. aestivum, durum, macha*
Oleosin*	Poaceae	Grasses	Stabilizes lipids	63	R.QAFQRT.P	Not unique to plants
					R.TPDYVEEAR.R	Poaceae
					R.QAFQRTPDY.V	Poaceae
					R.QAFQRTPDYVEE.A + Deamidated (NQ)	Poaceae
					R.QAFQRTPDYVEE.A + 2 Deamidated (NQ)	Poaceae
					R.QAFQRTPDYVEEA.R	Poaceae
					R.QAFQRTPDYVEEA.R + 2 Deamidated (NQ)	Poaceae
					R.QAFQRTPDYVEEA.R + Deamidated (NQ)	Poaceae
					R.QAFQRTPDYVEEAR.R	Poaceae
					R.QAFQRTPDYVEEAR.R	Poaceae
Non-specific lipid-transfer protein 2G and 2P*	*Triticum*	Wheat	Lipid transfer	63	K.DPTYGQYIR.S	*T. aestivum/Aegilops/durum*
					K.PSGECCGNLR.A	Triticeae

					R.AQQGCFCQYAK.D	BOP clade
					R.DTLTSCGLAVPHC.-	*T. aestivum & durum*
					R.DTLQSCGLAVPHC.- + Deamidated (NQ)	*T. aestivum & durum*
					S.AILSGAKPSGECCGNLR.A	*T. aestivum/Aegilops/durum*
					S.AILSGAKPSGECCGNLR.A	*T. aestivum/Aegilops/durum*
Germin-like protein 3-3*	BOP clade	Grasses	Defence	62	D.AGVVELLK.S	Not unique to plants
					R.VDAGVVELLK.S	BOP clade
					K.ALRVDAGVVELLK.S	BOP clade
					K.ALRVDAGVVELLK.S	BOP clade
Alpha-amylase inhibitor 0.28*	*Triticeae*	Wheat	Defence	56	R.SVYQELGVR.E	Triticeae
					R.CGDLSSMLR.S	Triticeae
					R.CGDLSSMLR.S + Oxidation (M)	Triticeae
Oxalate oxidase*	*Triticeae*	Wheat	Cell wall structure	54	E.AGVVELLK.S	Not unique to plants
					L.TLFGSDPPIPTPVLTK.A	*Hordeum vulgare***
Puroindoline-B*	*Triticeae*	Wheat	Defence	51	K.CNMGADCKFPSG.Y	Pooideae
					K.LSSCKDYVMER.C + Oxidation (M)	Triticeae
					R.AQSLPSKCNMGADCKFPSG.Y + Oxidation (M); Deamidated (NQ)	Pooideae
Gamma-gliadin*	*Triticinae*	Wheat	Major seed storage protein	51	L.VSIILPR.S	Not unique to plants
					S.LVSIILPR.S	Not unique to plants
					S.SLVSIILPR.S	Mesangiospermae
					F.LQQQMNPCK.N	Triticeae
					Q.GLGIIQPQQPAQLEGIR.S	Triticinae
Serine carboxypeptidase 2*	*Triticum*	Wheat	Enzyme	47	Y.ITVDEGAGR.S	*T. urartu & aestivum*
					Y.GASEELGAFR.V	Poales
					R.SLFYLLQEAPEDAQ.P	*Triticum/Aegilops*
					R.LPGQPAVDFDMYSGYITVDEGAGR.S + Oxidation (M)	*T. urartu & aestivum*
					R.LPGQPAVDFDMYSGYITVDEGAGR.S + Oxidation (M); Deamidated (NQ)	*T. urartu & aestivum*
Cysteine proteinase inhibitor 8 / Cysteine proteinase inhibitor 6*	Mesangiospermae	Flowering plants	Enzyme	47	S.GEQQVVSGMNYR.L	Mesangiospermae
					S.GEQQVVSGMNYR.L + Oxidation (M)	Mesangiospermae
Aspartic proteinase oryzasin-1	BOP clade	Grasses	Breakdow	43	N.YMNAQYFGEIGVGTPPQK.F + Oxidation (M); 2	Petrosaviidae

or Phytepsin*	n of storage proteins	Deamidated (NQ)	
		K.NYMNAQYFGEIGVGTPPQK.F + Oxidation (M); Deamidated (NQ)	Petrosaviidae
		K.VGEGAAAQCISGFTAMDIPPPR.G + Oxidation (M)	BOP clade

SI2. Plant proteins identified in the residue (#137337). Mascot scores are an indication of protein identification significance and are generated based on the probability of a random match. Scores are stated as -10*LOG10(P), where P is the absolute probability. *Proteins found expressed in the grain of the plant. ** Deamidation of N and D are not able to be to be unambiguously distinguished, so an assignment to *Hordeum vulgare* cannot be confidentially determined.
